# Indocyanine green angiography findings in patients with long-standing Vogt-Koyanagi-Harada disease: a cross-sectional study

**DOI:** 10.1186/1471-2415-12-40

**Published:** 2012-08-13

**Authors:** Felipe T da Silva, Carlos E Hirata, Viviane M Sakata, Edilberto Olivalves, Rony Preti, Sergio LG Pimentel, Andre Gomes, Walter Y Takahashi, Rogerio A Costa, Joyce H Yamamoto

**Affiliations:** 1 Uveitis Service, Department of Ophthalmology, Hospital das Clínicas, Universidade de São Paulo, São Paulo, SP, Brazil; 2 Retina Section, Department of Ophthalmology, Hospital das Clínicas, Universidade de São Paulo, São Paulo, SP, Brazil; 3 Division of Macula: Imaging & Treatment, Centro Brasileiro de Ciências Visuais, Belo Horizonte, MG, Brazil; 4 Rua Diana, 863 apto 91 J, São Paulo, SP, 05019-000, Brazil

**Keywords:** Angiography, Indocyanine green, Choroid, Diagnosis, Inflammation, Vogt-Koyanagi-Harada

## Abstract

**Background:**

To investigate indocyanine green angiography (ICGA) findings in patients with long-standing Vogt-Koyanagi-Harada (VKH) disease and their correlation with disease activity on clinical examination as well as with systemic corticosteroid therapy.

**Methods:**

Twenty-eight patients (51 eyes) with long-standing (≥6 months from disease onset) VKH disease whose treatment was tapered based only in clinical features were prospectively included at a single center in Brazil. All patients underwent standardized clinical evaluation, which included fundus photography, fluorescein angiography and ICGA. Clinical disease activity was determined based in the Standardization in Uveitis Nomenclature Working Group. Fisher exact test and logistic regression models were used for statistical analysis.

**Results:**

Disease-related choroidal inflammation on ICGA was observed in 72.5% (31 of 51 eyes). Angiographic findings suggestive of (choroidal and/or retinal) disease activity were not observed on FA. Clinically active disease based on clinical evaluation was observed in 41.2% (21 of 51 eyes). In these 21 eyes, disease-related choroidal inflammation on ICGA was observed in 76.2% (16 of 21 eyes); in the remaining eyes (without clinical active disease) disease-related choroidal inflammation on ICGA was observed in 70.0% (21 of 30 eyes). In respect to systemic corticosteroid therapy, 10 patients (18 of 51 eyes) were under treatment with prednisone. In these 10 (18 of 51 eyes) patients, disease-related choroidal inflammation on ICGA was observed in 83.3% (15 of 18 eyes); in the remaining patients (33 of 51 eyes) disease-related choroidal inflammation on ICGA was observed in 66.7% (22 of 33 eyes).

**Conclusion:**

ICGA findings suggestive of disease-related choroidal inflammation were observed in a considerable proportion of patients with long-standing VKH disease, independent of the inflammatory status of the disease on clinical examination or current use of systemic corticosteroid. Therefore, the current study reinforces the crucial role of ICGA to assist the management and treatment of patients with long-standing VKH disease.

## Background

Vogt-Koyanagi-Harada (VKH) disease is a T-cell altered immune process that is directed at the melanocytes [1]. The diagnosis is essentially based on the observation of bilateral diffuse choroiditis of acute onset following flu-like prodromic symptoms, and prompt treatment with systemic corticosteroid and/or immunosuppressant has been recommended. In spite of aggressive treatment during the acute phase of the disease, slowly progressive fundus depigmentation has been observed over time [2], even in the absence of detectable clinical signs of disease activity. There is increasing evidence that this slowly progressive fundus depigmentation is related to some disease activity undetected on regular clinical examination and is due to insufficient therapy; it does most probably not occur as part of the natural history of VKH disease [3].

In this sense, indocyanine green angiography (ICGA) may be an interesting ancillary tool. As the dye fluoresces in the near infrared wavelength, indocyanine green is particularly useful for imaging of the choroidal vascular compartment. In fact, the role of ICGA in detecting choroidal inflammation in patients with VKH disease has been investigated [4-7], mainly in European and Asian countries. Its relevance has been pointed out to either confirm elements already revealed by clinical examination or to determine the inflammatory status of the fundus and monitor VKH disease activity during follow-up, thus assisting the treatment process [4-7].

The use of ICGA for monitoring VKH disease activity, however, is not universally accepted [8,9]. Moreover, some of the ICGA findings suggestive of choroidal inflammation related to the disease may share angiographic features with those findings observed in patients under systemic corticosteroid treatment [10-13]. In the current report, we studied the ICGA findings in patients with long-standing VKH disease (i.e., patients who had more than 6 months from disease onset, regardless of the disease activity status determined on clinical examination). The possible correlation between ICGA findings and clinical disease activity as well as systemic corticosteroid therapy was investigated.

## Methods

### Subjects

Patients with long-standing VKH disease were prospectively enrolled in the study from March 2007 through October 2008 at a single center in Brazil (Uveitis Service, Hospital das Clínicas, Universidade de São Paulo, São Paulo, SP, Brazil) after written informed consent was obtained.

The diagnosis of VKH disease was based on the Revised Diagnostic Criteria proposed by the International Committee on Nomenclature [14]. Patients who were eligible for inclusion in the study had been treated during the acute phase with oral prednisone administered at 1.0-1.5 mg/kg daily, which was tapered according to disease activity over a period of time of at least 6 months. During this period disease activity was determined based on clinical examination and on fluorescein angiography (FA) findings.

The characterization of long-standing disease was based in the presence of a minimum time interval of 6 months from the onset of VKH disease, independently of the current clinical [phase] status of the disease. Patients with known allergy to fluorescein and/or indocyanine green dyes and media opacities that could prevent adequate fundus imaging were not eligible for study participation.

During the study period, disease activity was determined based on clinical evaluation, in accordance to the Standardization of Uveitis Nomenclature (SUN) Working Group [15]. Those eyes presenting cells in the anterior or posterior chamber or disc hyperemia, as well as any sign suggestive of posterior inflammation (for example, optic disc hyperfluorescence) on FA, were considered to have clinically active disease. Patients with clinically active disease by the time of initial study evaluation were treated with topical corticosteroid, associated or not with oral prednisone and/or immunosuppressant.

### Fundus photography and angiographic studies

Eligible patients underwent fundus photography (color and red-free) as well as angiographic studies (fluorescein and indocyanine green) using a conventional fundus camera system (TRC-50IX/IMAGEnet; Topcon Inc., Tokyo, Japan). Angiographic studies followed a standardized protocol previously described for the investigation of inflammatory eye diseases [16], and the eye with more pronounced fundus changes on clinical examination was selected for documentation of the initial transit phase of the dyes.

The ICGA data were evaluated by a retina specialist in a masked fashion. The following ICGA findings were categorically analyzed: 1) diffusely leaking choroidal vessels in the intermediate phase (“fuzzy vessels”), 2) diffuse choroidal hyperfluorescence in the late phase, and 3) hypofluorescent dark dots in the intermediate phase with later isofluorescence [6,7,16]. Those eyes presenting at least 2 of these findings were considered to have disease-related choroidal inflammation on ICGA.

### Ethics

This study protocol followed the statements of the Declaration of Helsinki and was approved by the Ethics in Research Committee analyzing research projects-CAPPesq from the Clinical Board of the Medical School of the University of São Paulo, under protocol #10121/2008.

### Statistical analysis

The correlation between the ICGA findings (i.e., disease-related choroidal inflammation) and disease activity on clinical examination (i.e., clinical activity) at the time of the angiographic study was evaluated using Fisher exact test. Logistic regression models were used to analyze the relationship between disease-related choroidal inflammation on ICGA and clinical activity as well as the use of systemic corticosteroid therapy. Data analysis and statistical tests were done using SPSS 15.0 (SPSS Science, Chicago, Illinois, USA) statistical software. The significance level was set at 0.05 for all tests.

## Results

Twenty-eight patients with long-standing VKH disease (51 eyes) were included in the study (in 5 patients, one eye was excluded due to media opacities). The ICGA findings were correlated with clinical activity and systemic corticosteroid therapy. The baseline characteristics are presented in Table 1.

**Table 1:** Demographics and clinical characteristics of the patients with Vogt-Koyanagi-Harada and long-standing disease

**Description**		
Number of patients (eyes)	28	(51)
Age (years), mean (± 1SD)	39.9	±13.0
Gender, male (%)	4	(14.3)
Race, n (%)		
White	12	(42.8)
Mestizo	10	(35.7)
Black	4	(14.3)
Asian/Oriental	2	(7.1)
Revised Diagnostic Criteria category [14], n (%)		
Complete	7	(25.0)
Incomplete	14	(50.0)
Probable	7	(25.0)
Clinical activity (SUN) [15], n (%)		
Remission^¶^	11	(39.3)
Inactive^§^	5	(17.8)
Active	12	(42.8)
Use of systemic medication, n (%)		
Prednisone only	5	(17.8)
Prednisone and immunosuppressant	5	(17.8)
Immunosuppressants only	3	(10.7)
None	15	(53.6)
Disease duration (in months), median (range)	91.5	(9–348)

^¶^Inactive disease for ≥3 months after discontinuing all treatments for eye disease.

^§^ Grade 0 cells.

**Figure 1:**
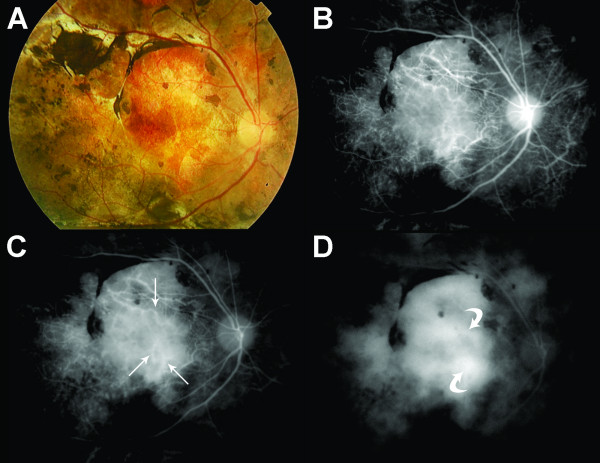
**Color fundus photography (A) as well as early, mid and late phase indocyanine green angiography (B,C,D, respectively) from a representative patient with Vogt-Koyanagi-Harada and long-standing disease.** Note “fuzzy vessels” (arrows) on early and mid phases ICGA, and “late diffuse hyperfluorescence” (curved arrows) on late phase of the exam.

At least one individual ICGA feature was observed in patients with long-standing VKH disease. The fuzzy vessels were observed in 80.4% (41 of 51 eyes), late diffuse hyperfluorescence in 78.4% (40 of 51 eyes), and dark dots in the intermediate phase with later isofluorescence in 19.6% (10 of 51 eyes). The presence of at least 2 of these ICGA features, thus characterizing disease-related choroidal inflammation on ICGA, was observed in 72.5% (37 of 51 eyes) (Figure 1). On FA, all eyes exhibited areas of hypofluorescence due to blockage interspersed with areas of hyperfluorescence due to window defects. Late hyperfluorescence due to staining of subretinal fibrovascular tissue was also observed in 37.3% (19 of 51 eyes). FA findings suggestive of (choroidal and/or retinal) disease activity were not observed.

In respect to clinical activity, 41.2% (21 of 51 eyes) were considered to have clinically active disease based on clinical evaluation. In all 21 eyes clinical signs indicative of some degree of anterior chamber inflammation (cells + to +++) were observed on clinical examination. Clinical signs suggestive of inflammation of the posterior segment were not observed on clinical examination.

In respect to systemic corticosteroid therapy, 10 patients (18 of 51 eyes) were under treatment with prednisone (with or without immunosuppressant agents). Three patients (5 of 51 eyes) were under treatment with immunosuppressant agents, and 15 patients (28 of 51 eyes) were using only topical treatment (Table 2).

**Table 2:** Correlation between disease-related choroidal inflammation on indocyanine green angiography, clinical disease activity and systemic corticosteroid therapy in 28 patients (51 eyes) with Vogt-Koyanagi-Harada and long-standing disease

	**N (eyes)**	**Disease-related choroidal inflammation on ICGA, n (%)**	***p***
**Total number of eyes**	51	37 (72.5)	
**Anterior chamber inflammation (cells)**			
Yes	21	16 (76.2)	0.626^†^
No	30	21 (70.0)	
**Systemic treatment at the time of ICGA**			
Prednisone	18	15 (83.3)	0.326^†^
Immunosuppressant	5	3 (60.0)	
No	28	19 (67.8)	

^†^Results of Fisher’s exact test.

The possible correlation of disease-related choroidal inflammation on ICGA with clinical activity and with systemic corticosteroid therapy was evaluated. In the 21 eyes with clinically active disease based on clinical evaluation disease-related choroidal inflammation on ICGA was observed in 76.2% (16 of 21 eyes). In the remaining 30 eyes (without clinical active disease) disease-related choroidal inflammation on ICGA was observed in 70.0% (21 of 30 eyes). In the patients (18 of 51 eyes) under systemic corticosteroid therapy, disease-related choroidal inflammation on ICGA was observed in 83.3% (15 of 18 eyes); in the remaining patients (33 of 51 eyes) disease-related choroidal inflammation on ICGA was observed in 66.7% (22 of 33 eyes).

## Discussion

The current study demonstrated that ICGA findings suggestive of disease-related choroidal inflammation was observed in a considerable proportion of patients with long-standing VKH disease whose treatment was tapered according to clinical and FA evaluation. Disease-related choroidal inflammation on ICGA was highly prevalent even among patients under systemic treatment as well as among those with no clinically detectable disease activity, thus confirming previous studies in European and Asian population and reinforcing the usefulness of ICGA to assist monitoring disease activity to better tailor treatment strategies.

Bouchenaki and Herbort described ICGA findings suggestive of choroidal involvement in acute VKH disease, which were less evident in the chronically evolving disease, and proposed ICGA as a useful tool to monitor the effect of steroid therapy [7]. Subtle ICGA findings in patients with VKH disease were subsequently shown to represent otherwise unnoticeable posterior segment disease-related activity [4-6]. These findings were described in the context of VKH disease during anterior segment relapses in the chronic phase and in patients tapering treatment in the convalescent phase of the disease. In both scenarios, intensification of immunosuppressive treatment resolved both, clinically apparent as well as the underlying disease process. Kawaguchi et al. coined the term partially treated VKH disease to describe patients in the convalescent phase whose treatment was intense enough to abolish anterior segment activity while leaving signs of choroidal involvement on ICGA, and who experienced progressive fundus depigmentation (sunset-glow fundus) [4]. In the current study, roughly three quarters of the eyes with long-standing VKH disease presented ICGA findings suggestive of disease-related choroidal inflammation regardless of the presence of activity on clinical examination. In fact, 70.0% of eyes demonstrated disease-related choroidal inflammation on ICGA without clinical evidence of disease activity, thus suggesting the presence of subclinical choroidal inflammation. Considering treatment regimen by the time of disease diagnosis, the majority of the patients included in this study had received early high-dose treatment. However, they were not systematically monitored with ICGA during the systemic treatment course, and clinical appraisal and fluorescein angiography were the only parameters used to taper treatment. Coupled with previous reports of better outcomes in more intensely treated patients, these observations provide additional evidence to support the possible role of ICGA in the adjustment of therapy at this stage of VKH disease [4,5,14-17]. The importance of ICGA guided management of VKH disease to meaningfully assess choroidal inflammation has been recently reinforced by Bouchenaki and Herbort [3]. These authors proposed that zero tolerance to subclinical choroidal inflammation could avoid irremediable evolution towards sunset glow fundus [3].

In the current study, fuzzy vessels and late diffuse hyperfluorescence were the most frequently findings observed on ICGA, thus suggesting some alteration in choroidal vessels’ permeability and choriocapillaris involvement [6]. Dark dots were considered only if they became isofluorescent (or mildly hyperfluorescent) in late-phase frames [6], and were the least observed ICGA finding in the current study. These observations are in line with previous histopathologic studies in patients with VKH and chronic disease in which a diffuse, non-granulomatous choroiditis accompanied by choriocapillaris involvement was described [1]. Additional findings identifiable on angiographic studies that have been correlated to serious ocular manifestations and/or severe choroidal inflammation at the acute uveitic stage of VKH disease [18-21], such as the presence of choroidal folds as reported by Wu et al. [18], were not identified in the current study.

**Figure 2:**
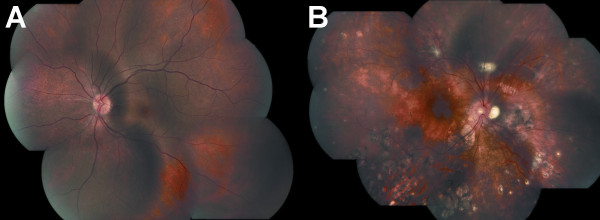
**Color fundus photography (photomontage) from patients with Vogt-Koyanagi-Harada and long-standing disease.** Mild (**A**) and severe disease (**B**) according to fundus-based disease severity grading (as per standardized analytic framework for ocular fundus alterations [22,23]).

Disease-related choroidal inflammation on ICGA was apparently more easily observed among those patients with milder fundus changes than in those with more severe fundus changes in the current study (Figure 2; Additional file 1: Table S3) [22,23]. It is possible that some eyes with fundus-based severe disease could also present subclinical choroidal inflammation that remains undetected on ICGA due to a combination of factors, such as the limited ability to identify ICGA signs of choroidal inflammation in a severely altered fundus with destruction and scarring of a considerable proportion of the choroidal stroma [6]. Naturally, the identification of the ICGA findings indicative of choroidal inflammation in patients with mild fundus changes seems an easier task. It should be noted that this study has limitations due to the relatively small number of patients and its cross-sectional design. In addition, the misinterpretation of the ICGA findings should always be considered in studies of this nature. Future studies, combining ICGA with other fundus imaging modalities such as fundus autofluorescence and enhanced depth imaging optical coherence tomography may facilitate our understanding of the choroidal involvement in patients with VKH disease [24,25], independent of the severity of disease associated fundus alterations.

## Conclusions

In the current study a considerable proportion of patients with long-standing VKH disease whose treatment was tapered based only on clinical and FA findings demonstrated ICGA findings suggestive of disease-related choroidal inflammation. Importantly, we have also demonstrated that these ICGA findings, which share some angiographic features with those associated with corticosteroid treatment, were observed independent of the use of systemic corticosteroid. Therefore, we herein provide additional evidence to support the use of ICGA for the detection of subclinical inflammation in patients with long-standing VKH disease, which may assist the management and treatment process of this entity, particularly in eyes with milder disease-related fundus changes.

## Competing interests

The authors declare that they have no competing interests.

## Authors’ contributions

FTBGCS made substantial contribution to acquisition, analysis, interpretation of data, in drafting and revising the manuscript critically; CEH participated in the design, interpretation of data and in revising the manuscript critically; VMS and EO participated in revising the manuscript critically; RCP, SLGP, AVG participated in the analysis, interpretation of data and in revising the manuscript critically; WYT participated in the design, in the analysis, interpretation of data and in revising the manuscript critically; RAC participated in the analysis, interpretation of data, in drafting and revising the manuscript critically; JHY participated in the design, coordination, analysis, interpretation of data, in drafting and revising the manuscript critically. All authors read and approved the final manuscript.

## Pre-publication history

The pre-publication history for this paper can be accessed here:

http://www.biomedcentral.com/1471-2415/12/40/prepub

## Supplementary Material

Additional file 1 **Table S3.** Correlation between indocyanine green angiography findings and fundus-based disease severity (as per standardized analytic framework for ocular fundus alterations [22,23]) in 28 patients (51 eyes) with Vogt-Koyanagi-Harada and long-standing disease. Click here for file
