# Supplementary material for: Indocyanine green angiography findings in patients with long-standing Vogt-Koyanagi-Harada disease: a cross-sectional study
Source: BMC Ophthalmol. 2012 Aug 13;12:40. doi: 10.1186/1471-2415-12-40 (PMC3480878; doi:10.1186/1471-2415-12-40)
Supplement: Additional file 1 — Table S3. Correlation between indocyanine green angiography findings and fundus-based disease severity (as per standardized analytic framework for ocular fundus alterations [22,23]) in 28 patients (51 eyes) with Vogt-Koyanagi-Harada and long-standing disease. [file 1471-2415-12-40-S1.doc]

**Supplementary material**

**Table 3.** Correlation between indocyanine green angiography findings and fundus-based disease severity (as per standardized analytic framework for ocular fundus alterations [22, 23]) in 28 patients (51 eyes) with Vogt-Koyanagi-Harada and long-standing disease.

| **Description** | **Total** | | | **Fundus grading^a^** | | | | | | | | | ***p*** |
| --- | --- | --- | --- | --- | --- | --- | --- | --- | --- | --- | --- | --- | --- |
|  |  |  |  | **Mild^¶^** | | | **Moderate^§^** | | | | **Severe^†^** | |  |
|  | **51 eyes** | | | **n (%)** | | | **n (%)** | | | | **n (%)** | |  |
| **Diffusely leaking choroidal vessels in the intermediate phase (“fuzzy vessels”)** | | | | | | | | | | | | | |
| Yes | 41 | (80.3) | 12 | | (92.3) | | | 16 | (84.2) | 13 | | (68.4) | 0.206 ^b^ |
| No | 10 | (19.7) | 1 | | | (7.7) | | 3 | (15.8) | 6 | | (31.6) |  |
| **Diffuse choroidal hyperfluorescence in the late phase** | | | | | | | | | | | | | |
| Yes | 40 | (78.4) | 12 | | (92.3) | | | 15 | (78.9) | 13 | | (68.4) | 0.237 ^b^ |
| No | 11 | (21.6) | 1 | | (7.7) | | | 4 | (21.1) | 6 | | (31.6) |  |
| **Hypofluorescent dark dots in the intermediate phase with later isofluorescence** | | | | | | | | | | | | | |
| Yes | 10 | (19.7) | 5 | | (31.5) | | | 2 | (10.5) | 3 | | (15.8) | 0.150 |
| No | 41 | (80.3) | 8 | | (68.5) | | | 17 | (89.5) | 16 | | (84.2) |  |
| **Disease-related choroidal inflammation (presence of 2 or more ICGA features)** | | | | | | | | | | | | | |
| Yes | 37 | (72.5) | 12 | | (92.3) | | | 14 | (73.7) | 11 | | (57.9) | 0.077^b^ |
| No | 14 | (27.5) | 1 | | (7.7) | | | 5 | (26.3) | 8 | | (42.1) |  |
| **^¶^** Mild or moderate diffuse fundus depigmentation and no other focal findings (nummular lesion in three quadrants or more, pigment clumps in three quadrants or more, any subretinal fibrosis) | | | | | | | | | | | | | |
| **^§^** Moderate or severe diffuse fundus depigmentation accompanied by one focal finding | | | | | | | | | | | | | |
| **^†^** Moderate or severe diffuse fundus depigmentation with at least two focal findings | | | | | | | | | | | | | |
| **^a^** Clinical graduation based on the analytic framework for ocular fundus alterations | | | | | | | | | | | | | |
| **^b^** results of likelihood ratio test | | | | | | | | | | | | | |
